# Elevated XIAP expression alone does not confer chemoresistance

**DOI:** 10.1038/sj.bjc.6605704

**Published:** 2010-05-18

**Authors:** J M Seeger, K Brinkmann, B Yazdanpanah, D Haubert, C Pongratz, O Coutelle, M Krönke, H Kashkar

**Affiliations:** 1 Institute for Medical Microbiology, Immunology and Hygiene, University of Cologne, Goldenfelsstrasse 19-21, 50935 Köln, Germany; 2 Center for Molecular Medicine Cologne (CMMC), University of Cologne, Joseph-Stelzmann-Str. 52, 50931 Köln, Germany; 3 Cologne Excellence Cluster on Cellular Stress Responses in Aging-Associated Diseases (CECAD), University of Cologne, Zülpicher Strasse 47, 50674 Köln, Germany

**Keywords:** XIAP, mitochondria, apoptosis, chemoresistance

## Abstract

**Background:**

In various tumour types, elevated expression of the X-linked inhibitor of apoptosis protein (XIAP) has been observed and XIAP targeting in diverse tumour entities enhanced the susceptibility to chemotherapeutic agents. Therefore, XIAP has been described and reviewed repeatedly as a chemoresistance factor in different tumour entities. However, rather than being an adverse prognostic marker, recent data suggest that elevated XIAP expression may be associated with a favourable clinical outcome. These somewhat conflicting findings, and the fact that in early studies XIAP suppressed apoptosis only when expressed transiently at levels far in excess of its physiological concentration, argue that the function of XIAP as an anti-apoptotic factor in tumour cells is both more complex and diverse than previously appreciated.

**Methods:**

To better understand the impact of long-term elevated XIAP expression on resistance to chemotherapy, we generated cell lines stably overexpressing XIAP. The role of mitochondria was examined by stable expression of Bcl2 or stable knockdown of second mitochondria-derived activator of caspase (SMAC) in combination with up- or downregulation of XIAP expression.

**Results:**

Our data show that long-term expression of XIAP at concentrations comparable to that in tumour cells (two- to five-fold increase) resulted in little or no resistance towards chemotherapeutic drugs. The XIAP overexpression only in conjunction with stable knockdown of a single XIAP-antagonising factor such as SMAC resulted in severe resistance to cytostatic agents demonstrating XIAP as a potent chemoresistance factor only in cells lacking functional XIAP regulatory circuits.

**Conclusion:**

Our results demonstrated that elevated XIAP expression alone cannot serve as a predictive marker of chemoresistance. Our data suggest that in order to predict the impact of XIAP on chemosusceptibility for a given tumour entity, the expression levels and functional states of all XIAP modulators need to be taken into account.

Each step of the apoptotic signalling cascade is under stringent control. Apoptotic signalling can be regulated at the apical point of the apoptotic cascade by controlling the translation of death-inducing signals into proteolytic activation or more critically by direct modulation of the proteolytic activity of caspases. The later is modulated by direct interaction of caspases with the X-linked inhibitor of apoptosis protein (XIAP). The XIAP is the only cellular protein that has evolved to potently inhibit the enzymatic activity of mammalian caspases at both the initiation phase (casp-9) and the execution phase (casp-3 and -7) of apoptosis (Deveraux *et al*, 1997). The anti-apoptotic activity of XIAP is in turn controlled by the so-called second mitochondria-derived activator of caspases (SMACs), which inhibits XIAP by direct binding (Vaux and Silke, 2003). The SMAC is a mitochondrial protein that is released during apoptosis along with cytochrome *c* (cyt *c*) and other mitochondrial apoptogenic factors. Once in the cytosol, SMAC binds to XIAP and disrupts its caspase-inhibiting activity thereby restoring the apoptotic machinery.

Since the discovery of XIAP in the second half of the 1990s, our understanding of this unique IAP has progressed rapidly, including a detailed structural and mechanistic view of its activity in addition to abundant cell biological data (Eckelman *et al*, 2006). According to this data, XIAP is thought to render tumour cells resistant to multi-agent chemotherapy through its ability to inhibit caspases, and, on this basis, has been proposed as an important adverse prognostic factor responsible for tumour chemoresistance (Schimmer *et al*, 2006). At odds with these findings, however, elevated XIAP expression has recently been shown to be associated with a favourable clinical outcome (Ferreira *et al*, 2001; Seligson *et al*, 2007; Hwang *et al*, 2008).

The critical point in determining the precise role of XIAP in drug resistance besides its expression level is the degree and involvement of cellular regulatory circuits directly or indirectly controlling XIAP function. Indeed, in the initial investigations, XIAP was capable of suppressing apoptosis only when transiently expressed at levels far in excess of physiological values and disregarding the mitochondrial check points representing the central regulatory machinery in controlling XIAP action (Srinivasula and Ashwell, 2008). The physiological relevance of these observations is therefore limited. To better understand the impact of long-term elevated XIAP expression on resistance to chemotherapy, we generated cell lines stably expressing XIAP. The role of mitochondria was examined by the stable expression of Bcl2 or stable knockdown of SMAC in combination with up- or downregulation of XIAP expression. Our data show that increased XIAP expression alone does not induce chemoresistance and that the anti-apoptotic function of XIAP is secondary to defective mitochondrial responses to apoptotic stimuli.

## Materials and methods

### Cell lines and cell culture

HeLa, HeLa-Bcl2, and HeLa-mycXIAP (Kashkar *et al*, 2005, 2007) cell lines were cultured in DMEM supplemented with 10% foetal calf serum, 2 mM L-glutamine, 100 *μ*g ml^−1^ streptomycin, and 100 units ml^−1^ penicillin (Biochrom, Berlin, Germany). Cytostatic agents were purchased from Sigma (Deisenhofen, Germany), except for staurosporine (STS) (Alexis, Lausen, Switzerland). Caspase activity was blocked by co-treatment of cells with 20 *μ*M Z-VAD(Ome)-FMK (Alexis, Grünberg, Germany).

### Sample preparation, immunoblotting, and immunoprecipitation

Whole-cell extracts were prepared by cell lysis in CHAPS buffer. Poly(ADP-ribose) polymerase (PARP) cleavage was assessed after incubation of pellets in urea-extraction buffer. Cytosolic extracts were prepared in buffer A as described. Rabbit polyclonal antiserum for human Bax and mouse monoclonal antibodies for XIAP, Bcl2, PARP, active Bax (clone 6A7), and cyt *c* were from BD Laboratories (Heidelberg, Germany); mouse anti-SMAC antibody was from Cell Signalling Technology (Beverly, MA, USA). Active Bax was detected with anti-Bax antibody (clone 6A7) (Kashkar *et al*, 2002, 2005).

### siRNA and lentiviral gene transfer

The pLenti6/V5DEST XIAPshRNA–, SMACshRNA–, or scrshRNA-expressing vectors was created using LR recombination (ViraPower Lentiviral expression system, Invitrogen, Karlsruhe, Germany). Stable cell lines were generated by blasticidin selection (Invitrogen) as described (Kashkar *et al*, 2006).

### Cell viability

Cells (10^4^ per well) were incubated in 96-well plates with cytostatic agents as indicated. Cell viability was assessed by the XTT test (Cell Proliferation Kit II, Roche Applied Sciences, Mannheim, Germany) or crystal violet assay system (CVS). For XTT, absorbance was measured with an ELISA reader (at 450 nm; reference at 620 nm). For CVS, cells were washed in PBS, stained with crystal violet (0.2% w/v in 2% EtOH), and dissolved in 0.2 M sodium citrate and 100% EtOH (1 : 1 v/v). The colorimetric measurement was carried out at 595 nm (Tecan GENious Pro; ThermoFischer Scientific, Langenselbold, Germany). Data are means of at least three different experiments in triplicate. Blank absorbance was subtracted from the samples, and the difference was expressed as ‘% cell viability’ (100% in untreated cells). Clonogenicity was determined as described (Vogler *et al*, 2007).

## Results

### Elevated XIAP expression does not confer chemoresistance

**Figure 1:**
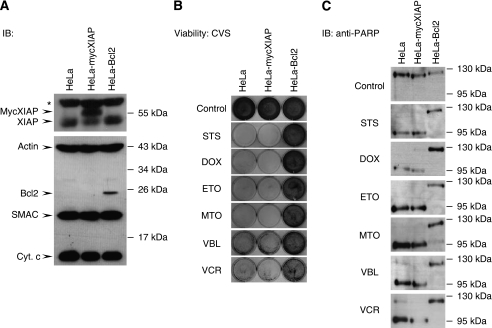
Cytostatic agent-induced cell death. (**A**) X-linked inhibitor of apoptosis protein (XIAP), actin, Bcl2, second mitochondria-derived activator of caspase (SMAC), and cytochrome *c* (cyt *c*) were detected in total cell extracts of HeLa, HeLa-mycXIAP, and HeLa-Bcl2 by western blotting. (**B** and **C**) HeLa, HeLa-mycXIAP, and HeLa-Bcl2 cell lines were treated for 24 h with staurosporine (STS, 0.5 *μ*M), doxorubicin (DOX, 5 *μ*M), etoposide (ETO, 100 *μ*M), mitoxantrone (MTO, 5 *μ*M), vinblastine (VBL, 50 nM), and vincristine (VCR, 50 nM). Viability was assessed by crystal violet staining (CVS). Poly(ADP-ribose) polymerase (PARP) cleavage was detected in nuclear extracts using mouse anti-PARP antibody (**C**). ^*^Indicates nonspecific bands recognised by anti-XIAP antibody. IB, immunoblotting.

To characterise the role of XIAP as a chemoresistance factor, a HeLa cell line stably expressing myc-tagged XIAP (mycXIAP) was established (Figure 1A). The cytoprotective potency of XIAP was evaluated in the HeLa-mycXIAP cell line after treatment with chemotherapeutic agents, including STS, doxorubicin (DOX), etoposide (ETO), mitoxantrone (MTO), vinblastine (VBL), and vincristine (VCR), and compared to unmodified HeLa cells as well as a HeLa cell line stably expressing Bcl2 (HeLa-Bcl2) as an established chemoresistance factor (Reed, 1995; Kashkar *et al*, 2005; Letai, 2008) (Figure 1A). Unexpectedly, XIAP expression had no cytoprotective effect nor did XIAP expression confer an anti-apoptotic effect towards cytostatic agents (Figure 1B and C). All cytostatic agents exerted comparable cytotoxic effects in HeLa and HeLa-mycXIAP cell lines in various cytotoxicity assays including CVS (Figure 1B), XTT, trypan blue exclusion, and clonogenicity assays (Supplementary Figure S1). Cleavage of PARP was analysed to monitor the ongoing apoptotic process and to demonstrate a functional apoptotic cascade in HeLa and HeLa-mycXIAP cell lines upon cytostatic treatments (Figure 1C). In contrast to XIAP expression, stable Bcl2 expression completely abrogated the apoptotic capability of cells in response to cytostatic drugs (Figure 1B and C and Supplementary Figure S1).

### Caspase-independent mitochondrial SMAC release in response to cytostatic agents diminishes the anti-apoptotic potential of XIAP

**Figure 2:**
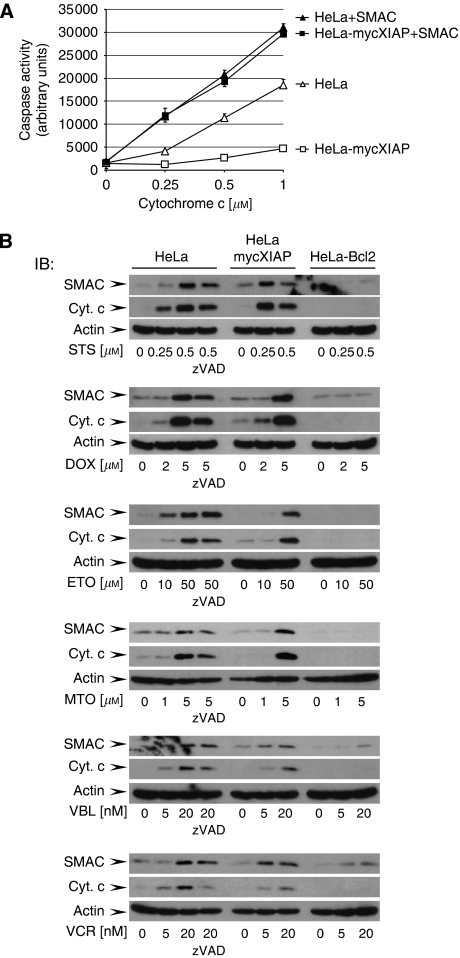
Cytostatic agents induce caspase-independent second mitochondria-derived activator of caspase (SMAC) release. (**A**) Cytosolic extracts of HeLa and HeLa-mycXIAP cells were prepared by incubating with increasing amount of cytochrome *c* (cyt *c*; 0, 0.25, 0.5, and 1 *μ*M) and dATP with or without SMAC N7 peptide for 15 min at 30°C. Relative casp-3 activity was measured using 100 *μ*M Ac-DEVD-AFC as arbitrary units. Values represent means±s.d. from at least three experiments in triplicate. (**B**) HeLa, HeLa-mycXIAP, and HeLa-Bcl2 cells (all 10^6^) were treated with staurosporine (STS; 0, 0.25, and 0.5 *μ*M), doxorubicin (DOX; 0, 2, and 5 *μ*M), etoposide (ETO; 0, 10, and 50 *μ*M), mitoxantrone (MTO; 0, 1, and 5 *μ*M), vinblastine (VBL; 0, 5, and 20 nM), and vincristine (VCR; 0, 5, and 20 nM) for 12 h. SMAC and cyt *c* were detected in cytosolic extracts by western blotting. Actin was served as loading control. Caspase activity in cytostatic drug-induced cytchrome c/SMAC release was examined in HeLa cells by z-VAD-FMK (20 *μ*M) co-treatment. IB, immunoblotting.

To investigate the inhibitory potency of stably expressed XIAP on caspase activity in the HeLa-mycXIAP cell line, cytosolic extracts of intact HeLa cells were prepared and caspase activity was initiated by exogenously added cyt *c* and dATP (Kashkar *et al*, 2003). In contrast to the parental HeLa cells, casp-3 activity was significantly impaired in cytosolic extracts derived from HeLa-mycXIAP cells, demonstrating the caspase inhibitory potency of stably expressed mycXIAP. As the sensory centres of cytotoxic stresses, the mitochondria promote caspase activity by releasing pro-apoptotic factors, including cyt *c* and SMAC. Once released into the cytosol, SMAC interacts with XIAP to release XIAP-mediated inhibition of casp-3. Accordingly, in cytosolic extracts of HeLa and HeLa-mycXIAP cells, addition of the synthetic SMAC agonist (N7 peptide) enhanced casp-3 activity initiated by cyt c/dATP (Figure 2A). Detailed analyses of the mitochondrial apoptotic pathway in HeLa, HeLa-mycXIAP, and HeLa-Bcl2 cell lines showed that all tested cytostatic agents were capable of initiating the mitochondrial release of cyt *c* and SMAC in HeLa and HeLa-mycXIAP cells, but not in the HeLa-Bcl2 cells with blocked mitochondria (Figure 2B). The release of the mitochondrial pro-apoptotic factors in response to cytostatic drug treatment was predominantly a caspase-independent process as demonstrated by pretreatment with the universal caspase inhibitor z-VAD (Figure 2B). In contrast to Bcl2, XIAP was not able to prevent the cytostatic agent-induced mitochondrial release of SMAC (Figure 2B).

**Figure 3:**
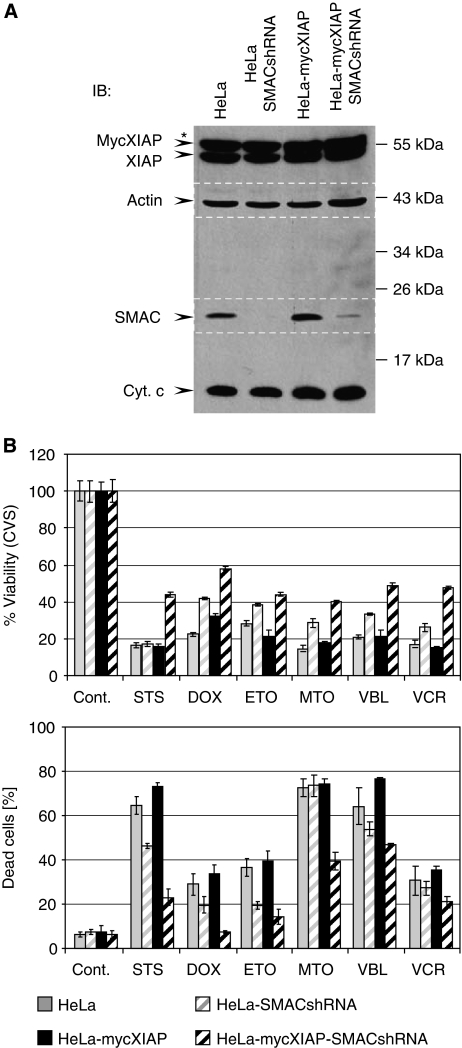
Knockdown of second mitochondria-derived activator of caspase (SMAC) promotes resistance to cytostatic agents and facilitates anti-apoptotic function of X-linked inhibitor of apoptosis protein (XIAP). (**A**) XIAP, actin, SMAC, and cytochrome *c* (cyt *c*) were detected in total cell extracts of HeLa, HeLa-SMACshRNA, HeLa-mycXIAP, and HeLa-mycXIAP-SMACshRNA cells by western blotting for XIAP, cyt *c*, actin (reprobed and merged), and SMAC (reprobed and merged). (**B**) Cells were treated for 24 h with staurosporine (STS, 0.5 *μ*M), doxorubicin (DOX, 5 *μ*M), etoposide (ETO, 100 *μ*M), mitoxantrone (MTO, 5 *μ*M), vinblastine (VBL, 50 nM), and vincristine (VCR, 50 nM). The percentage of viable cells was determined by crystal violet staining (CVS) and cell death by trypan blue exclusion. Values represent means±s.d. of at least three experiments carried out in triplicate. ^*^Indicates nonspecific bands recognised by anti-XIAP antibody. IB, immunoblotting.

**Figure 4:**
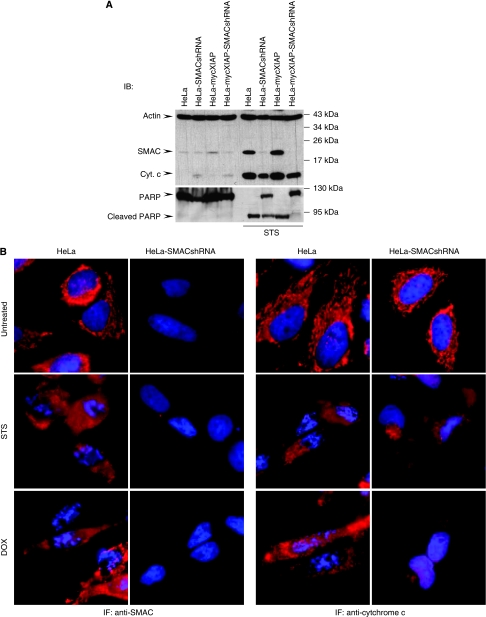
Knockdown of second mitochondria-derived activator of caspase (SMAC) does not influence mitochondrial release of cytochrome *c* (cyt *c*). (**A**) Cells were treated with staurosporine (STS) (0.5 *μ*M) for 12 h. SMAC and cyt *c* were detected in cytosolic extracts and poly(ADP-ribose) polymerase (PARP) cleavage in nuclear extracts by western blotting. Actin served as a loading control. (**B**) Immunofluorescence (IF) analysis of SMAC and cyt *c* release after STS and doxorubicin (DOX) treatment in HeLa and HeLa-SMACshRNA cell lines. Cells were treated with STS (0.5 *μ*M) or DOX (5 *μ*M) for 8 h. SMAC and cyt *c* were detected with Alexa Fluor 568-conjugated antibodies (red). Nuclei were co-stained with Hoechst 33258. IB, immunoblotting.

**Figure 5:**
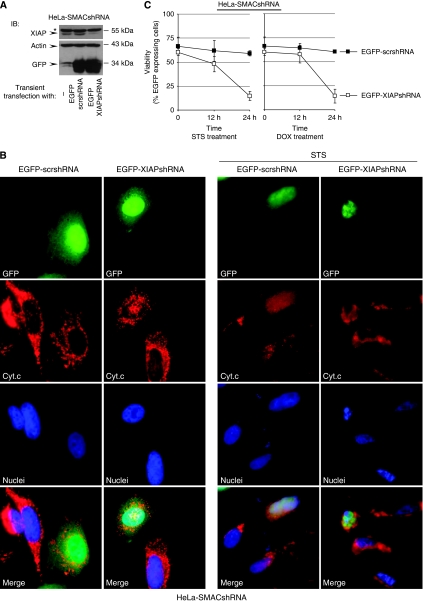
X-linked inhibitor of apoptosis protein (XIAP) knockdown restores inefficient apoptosis in HeLa-SMACshRNA cells. HeLa-SMACshRNA cells were transfected with EGFP-XIAPshRNA or EGFP-scrshRNA expression plasmids. (**A**) After 48 h, cell extracts were prepared and XIAP, actin, and green fluorescent protein (GFP) were detected by western blotting. (**B**) Cells were treated with staurosporine (STS, 0.5 *μ*M) for 8 h. cytochrome *c* (cyt *c*) was detected with Alexa Fluor 568-conjugated antibodies (red). Nuclei were co-stained with Hoechst 33258. Transfected cells co-expressed enhanced GFP (EGFP) (green). (**C**) Transfected cells (**A**) were treated with STS or doxorubicin (DOX) for 12 and 24 h. Viability was microscopically evaluated in >300 EGFP-expressing cells. The percentage of viable EGFP expressing cells was calculated relative to the total cell number (Kashkar *et al*, 2005). Values represent means±s.d. of two experiments carried out in triplicate. ^*^Indicates nonspecific bands recognised by anti-XIAP antibody. IB, immunoblotting.

If, indeed, the mitochondrial release of SMAC was responsible for the neutralisation of XIAP, then downregulation of SMAC should restore the caspase-inhibitory function of XIAP and increase resistance towards cytostatic agents. To address this issue, we stably downregulated SMAC expression using small hairpin RNA (shRNA) targeting SMAC mRNA (Kashkar *et al*, 2006) (Figure 3A). As expected, HeLa-SMACshRNA cells showed a markedly reduced susceptibility to chemotherapeutic treatments compared with their parental counterparts, HeLa and HeLa-mycXIAP, and this was even more pronounced in the presence of XIAP overexpression shown in HeLa-mycXIAP-SMACshRNA cells (Figure 3B and Supplementary Figure S2). Altogether, these data demonstrate a regulatory role of SMAC during cytostatic agent-induced cell death. Importantly, the SMAC knockdown did not cause any changes in cyt *c* release after cytostatic drug treatment that could account for the reduced apoptotic activity (Figure 4). Rather, the knockdown of SMAC in HeLa-SMACshRNA cells resulted in the blockade of caspase activity as illustrated by incomplete PARP cleavage (Figure 4A and Supplementary Figure S3) and a failure of nuclear fragmentation (Figure 4B). Inhibition of caspase activity was more pronounced in XIAP-expressing HeLa-mycXIAP-SMACshRNA cells as shown by complete blockade of PARP cleavage (Figure 4A and Supplementary Figure S3). The antagonistic action of SMAC and XIAP was confirmed by XIAP knockdown in HeLa-SMACshRNA cells (Figure 5). HeLa-SMACshRNA cells were transiently transfected with DNA constructs containing XIAPshRNA or scrshRNA expression cassettes in addition to co-expressing EGFP to visualise the transfected cells (Figure 5A). Immunofluorescence analysis revealed nuclear fragmentation as a sign of the ongoing apoptotic process after STS treatment in HeLa-SMACshRNA cells transiently transfected with EGFP-XIAPshRNA, but not in untransfected cells or cells transfected with EGFP-scr-RNA (Figure 5B). Correspondingly, the viability of HeLa-SMACshRNA cells depleted of XIAP was also reduced in response to STS or DOX treatments (Figure 5C). It is important to note that chemoresistance caused by loss of SMAC expression was only reversed when XIAP was downregulated, but not by knockdown of other members of the IAP protein family such as cIAP1 and cIAP2 (Supplementary Figure S4). Altogether, these data demonstrate the specific interplay between XIAP and SMAC in modulating caspase activity and determining the drug-resistant phenotype.

## Discussion

As a prominent member of the IAP protein family with the most potent caspase inhibitory capacity (Eckelman *et al*, 2006), XIAP, is regarded as a powerful chemoresistance factor. However, in the initial investigations, using transient overexpression systems, XIAP was capable of suppressing apoptosis only at levels far in excess of its physiological concentrations. Since then, XIAP has also been shown to be crucially involved in several non-apoptotic signalling cascades. Therefore, its description as ‘X-linked inhibitor of apoptosis’ has recently been called into question, suggesting that the anti-apoptotic functions ascribed to XIAP may not reflect the true nature of its normal biological activities (O'Riordan *et al*, 2008; Srinivasula and Ashwell, 2008). In agreement with this, long-term expression of XIAP at concentrations comparable to that in tumour cells (two- to five-fold increase) (Kashkar *et al*, 2003) resulted in little or no resistance towards chemotherapeutic drugs (Figure 1).

Several factors including SMAC, Omi, XAF1 (XIAP-associated factor 1), and several IAP-binding motif (IBM)-containing proteins, including polypeptide chain-releasing factor (GSPT1) and Checkpoint kinase 1 (Chk1), have critical roles in controlling XIAP functions (LaCasse *et al*, 2008; Srinivasula and Ashwell, 2008). However, previous studies describing the function of XIAP in cell-free systems using recombinant proteins (Deveraux *et al*, 1997, 1998), yeast models (Silke *et al*, 2001, 2002), or transient-overexpression studies in intact cells (Deveraux *et al*, 1998; Silke *et al*, 2001) have disregarded the cellular regulatory mechanisms controlling XIAP function. Strikingly, here we show that stable knockdown of a single XIAP-antagonising factor such as SMAC resulted in severe resistance to cytostatic agents on XIAP overexpression (Figures 2, 3, 4, 5), demonstrating XIAP as a potent chemoresistance factor in cells lacking functional XIAP regulatory circuits.

Interestingly, several recent studies failed to demonstrate a correlation between XIAP expression and clinical outcomes, although some reported an association between XIAP expression and favourable clinical outcomes (Ferreira *et al*, 2001; Liu *et al*, 2001; Carter *et al*, 2003; Seligson *et al*, 2007; Hwang *et al*, 2008). Our data suggest that, in order to predict the impact of XIAP on chemosusceptibility for a given tumour entity, the expression levels and functional states of all XIAP modulators need to be taken into account. That the finely tuned balance between XIAP and its antagonists is critical in determining the clinical outcome in cancer patients was demonstrated in an analysis of 187 gastric adenocarcinomas in which the expression levels of XIAP and its antagonising factors including SMAC and XAF1 were examined. The XIAP, SMAC, and XAF1 individually were not associated with disease-specific survival. However, patients showing high expression levels of XIAP and low expression of XAF1 had significantly poorer survival when compared with other groups (Shibata *et al*, 2008). Together these data suggest that XIAP-targeting drugs could be particularly effective in tumours in which XIAP function is unopposed because the regulatory mechanisms are inactivated. Such tumour targets are already known, including Hodgkin's lymphoma, CLL, and melanoma. The use of SMAC mimetics to sensitise such malignancies for chemotherapy offers an exciting new treatment option.
