# Supplementary material for: Elevated XIAP expression alone does not confer chemoresistance
Source: Br J Cancer. 2010 May 18;102(12):1717–23. doi: 10.1038/sj.bjc.6605704 (PMC2883704; doi:10.1038/sj.bjc.6605704)
Supplement: Supplementary Figures S1–S4 [file 6605704x1.pdf]

S1

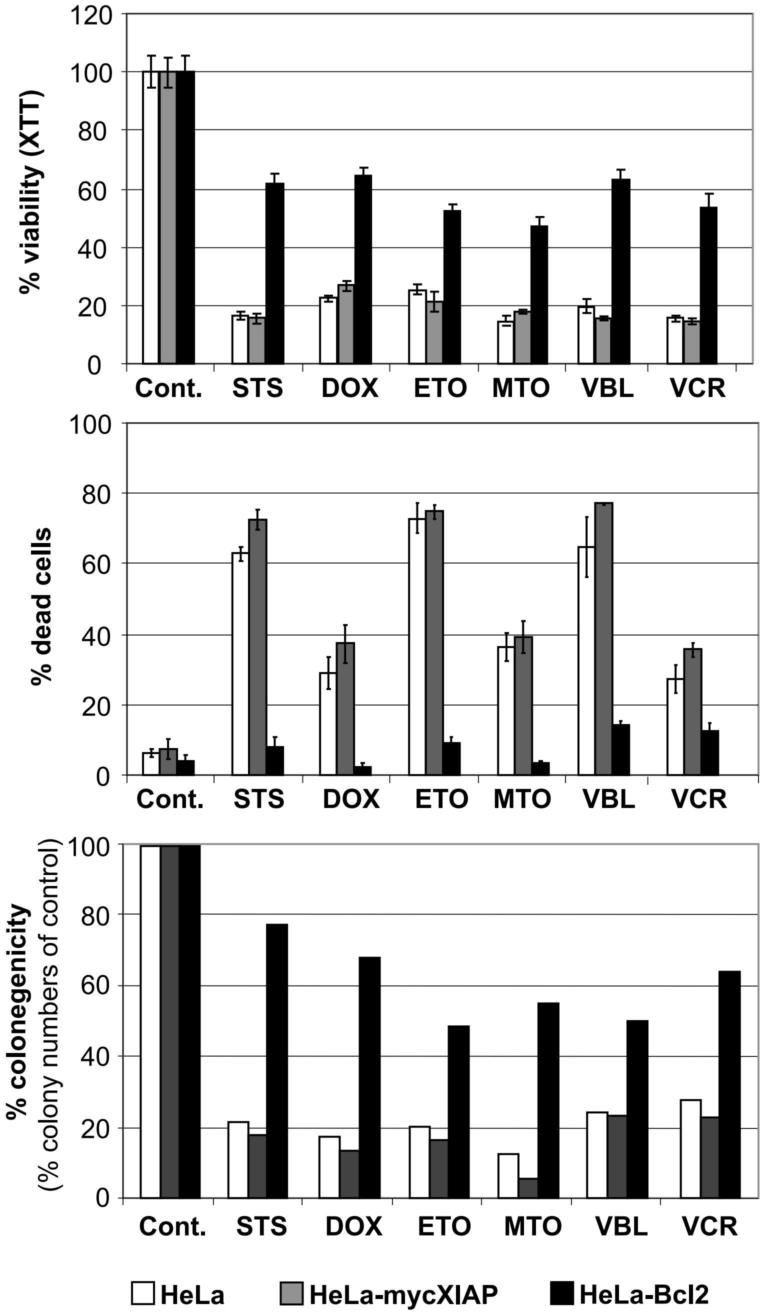

Supplementary Fig. 1. (S1) HeLa, HeLa-mycXIAP and HeLa-Bcl2 cell lines were left untreated or treated for 24 hours with staurosporine (STS, 0.5 µM), doxorubicin (DOX, 5 µM), etoposide (ETO, 100 µM), mitoxantrone (MTO, 5 µM), vinblastine (VBL, 50 nM), and vincristin (VCR, 50 nM). Viable cell number was determined using XTT. Cell death was determined by trypan blue exclusion. The experimental values represent mean ± SD values from at least 3 individual experiments performed in triplicate. In order to examine the colonogenicity 1000 cells were seeded in 12-well plates and incubated o. n. After 1h treatment with STS (0.5 µM), DOX (5 µM), MTO (5 µM), VBL (0.1 µM), VCR (0.1 µM) or ETO (100 µM) the cells have been washed twice with PBS and cultured for 14-21 days until colonies have formed. The counted colonies are shown as % of colonies formed by untreated cells.

S2

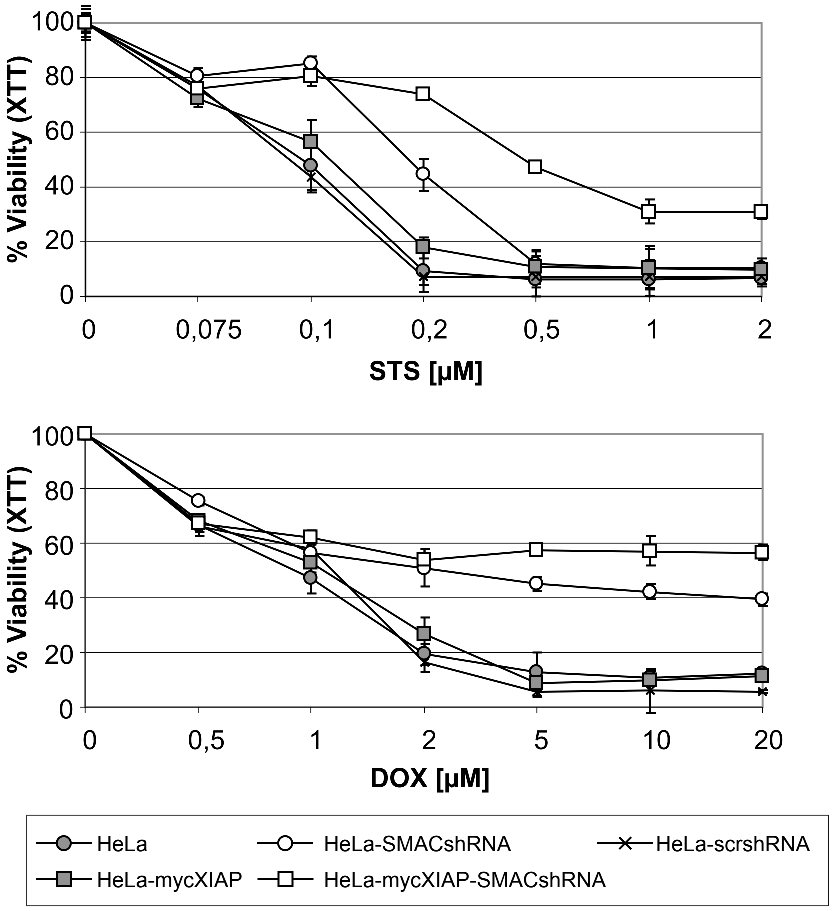

Supplementry Fig. 2. (S2) SMAC knock-down promotes resistance against cytostatic agents and facilitates XIAP anti-apoptotic function. Cells were left untreated or treated for 24 hours with increasing concentrations of STS or DOX. The percentage viable cell number was determined using an XTT assay.

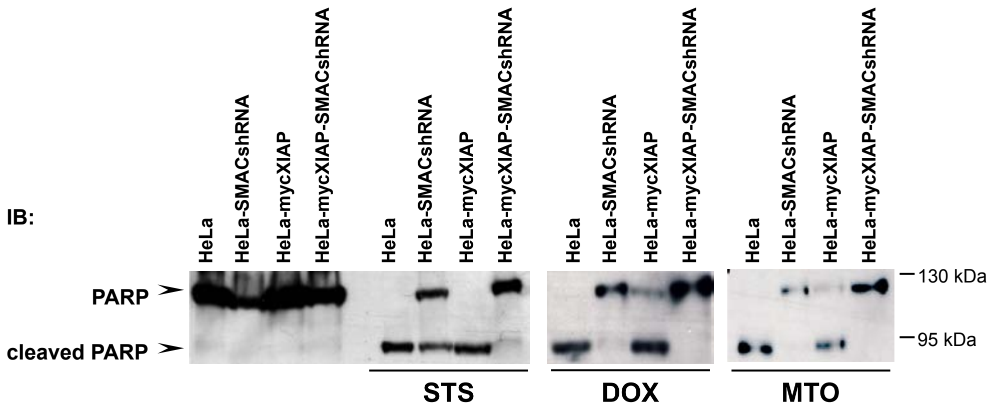

Supplementary Fig. 3. (S3) SMAC is the central determinant of chemosusceptibility. Cells were treated with STS, DOX or MTO for 12 h. PARP cleavage was detected in nuclear extracts using mouse anti-PARP antibody.

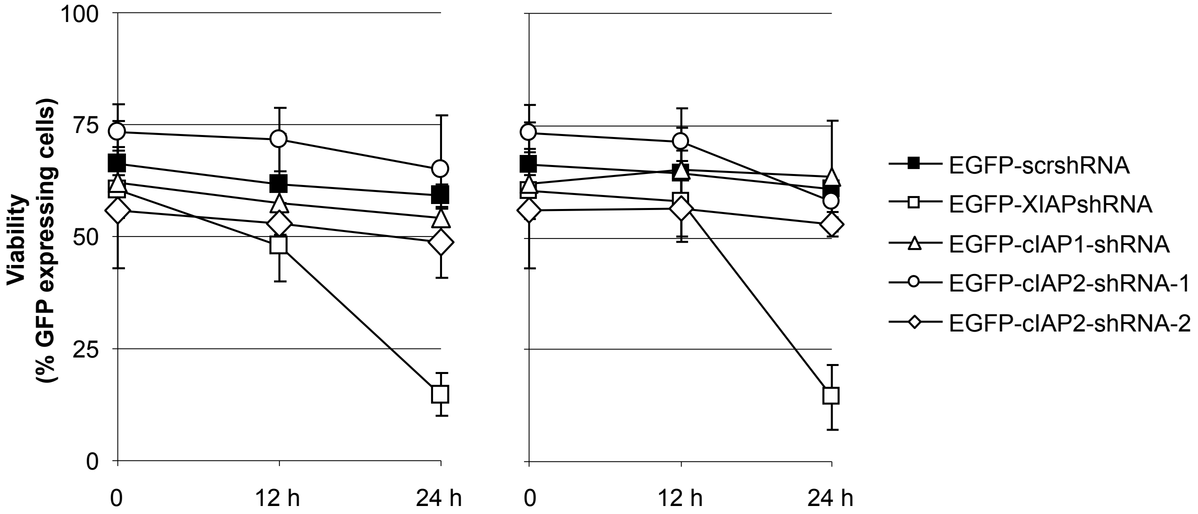

Supplementary Fig. 4. (S4) XIAP knock-down restores inefficient apoptosis in HeLa-SMACshRNA cells. HeLa-SMACshRNA cells were transiently transfected with DNA constructs encoding EGFP together with XIAPshRNA, scrshRNA or validated cIAP1-shRNA or two independent cIAP2-shRNAs expression cassettes. Transfected cells were treated with STS or DOX for 12 and 24 h. Viability was microscopically determined by evaluating >300 EGFP expressing cells. The percentage of viable EGFP expressing cells was calculated relative to the total cell number {Kashkar, 2005 #19). The values represent mean ± SD values from 2 individual experiments performed in triplicate. Asterisk indicates nonspecific bands recognized by anti-XIAP antibody.
